# Is Dealing with Climate Change a Corporation’s Responsibility? A Social Contract Perspective

**DOI:** 10.3389/fpsyg.2016.01212

**Published:** 2016-08-18

**Authors:** Kerrie L. Unsworth, Sally V. Russell, Matthew C. Davis

**Affiliations:** ^1^Leeds University Business School, University of LeedsLeeds, UK; ^2^Sustainability Research Institute, School of Earth and Environment, University of LeedsLeeds, UK

**Keywords:** corporate social responsibility, environmental responsibility, climate change, pro-environmental behavior, behavior change, micro CSR

## Abstract

In this paper, we argue that individuals – as members of society – play an important role in the expectations of whether or not companies are responsible for addressing environmental issues, and whether or not governments should regulate them. From this perspective of corporate social responsibility as a social contract we report the results of a survey of 1066 individuals. The aim of the survey was to assess participants’ belief in anthropogenic climate change, free-market ideology, and beliefs around who is responsible for dealing with climate change. Results showed that both climate change views and free market ideology have a strong effect on beliefs that companies are responsible for dealing with climate change and on support for regulatory policy to that end. Furthermore, we found that free market ideology is a barrier in the support of corporate regulatory policy. The implications of these findings for research, policy, and practice are discussed.

## Introduction

Corporate social responsibility (CSR) is based on the assumption that, at any given point in time, there is a social contract between an organization and society in which the organization has not only economic and legal responsibilities but also ethical and philanthropic responsibilities (Carroll, 1991, 1999; Donaldson and Dunfee, 1994, 1999, 2000). Building on Carroll’s (1991, 1999, 2000, 2004) work, Dahlsrud (2008) conducted a review of CSR definitions and suggested that the majority of definitions include at least two of the following dimensions (although they very rarely have all of them): societal concerns, interacting with stakeholders and social contract, an economic upside, environmental issues, and actions performed voluntarily. As such, we define CSR as a societal expectation, based on stakeholder perspectives (Carroll, 2004), that organizations should address economic, legal, ethical, and discretionary responsibilities. Within this research we are interested in two areas of CSR that Dahlsrud (2008) found to be less represented in current definitions – that of environmental issues and voluntary vs. forced action – and explore both what the social contract is surrounding these dimensions and how belief in anthropogenic climate change and free market ideology affect those societal views.

Underlying our research is the need to examine the social contract on which CSR rests (Donaldson and Dunfee, 1994, 1999, 2000). If society’s expectations change, then CSR must as well or else it will face a perceived violation of the social contract. Pinkston and Carroll (1996) note that the demands that the public place on corporations do change; yet most of the research on CSR considers how the corporations themselves see their responsibilities or, at most, how their customers see their responsibilities (e.g., Mohr et al., 2001; Russell et al., 2016), and not how society more broadly views them. Alongside many others, we argue that because CSR is a social contract there is a need to understand not only what corporations and academics believe CSR to be, but also what society believes CSR to be and thus how it is operationalized in practice (Sacconi, 2006). We do so in the particular realm of environmental responsibility.

Dahlsrud’s (2008) analysis shows that the environmental dimension was the least common in definitions of CSR. This is surprising given the urgent need for organizations to address environmental issues and climate change (Pinkse and Kolk, 2009; Winn et al., 2011). Specifically, there is clear evidence that climate change and its effects are having deepening impacts across the globe and governments are contemplating the use of regulations to mitigate against further climate change. In this research we explore climate change as a CSR issue and question whether climate change should be included in the operationalization of CSR. As we are interested in the social contract, we specifically investigate whether society considers that dealing with climate change is something that a corporation should be concerned with.

The second key focus of this research is on voluntary action. Although some have suggested that CSR must be voluntary for it to be considered CSR (Dahlsrud, 2008), it was not included in all definitions reviewed by Dahlsrud (2008) and therefore is debated rather than definitive. Instead, we follow Carroll’s (1991) framework that includes not only completely discretionary and ethical (in other words, voluntary but morally urged) CSR but also legally and economically motivated CSR, such as not polluting. Our research, therefore, allows us to explore whether climate change is seen, in the social contract, as being in the discretionary category of CSR actions, or whether the social contract around climate change action is such that it be mandated by government and thus fall in the legal category of Carroll’s framework.

### Societal Expectations Regarding a Corporation’s Duty to Deal with Climate Change

The first part of our research is with the social contract itself; in other words, does the community view the social contract with corporations as encompassing climate change action and, if it is not discretionary (i.e., that they do see dealing with climate change as being the corporation’s duty), then to what extent do they believe it should be legally mandated? We argue that it is important to understand how the community views the social contract with corporations, particularly with regard to the specific concept of climate change, because only then will we have an understanding of expectations from both sides of the social contract.

Marketing research has long looked at society’s expectations of corporations with regard to CSR, by examining consumer’s expectations. For example, Mohr et al. (2001) conducted in-depth interviews with 48 people and found that over half desired moderately high or high levels of CSR from companies. Maignan (2001) surveyed over 300 people from Germany, France, and USA; she found that consumers in France and Germany believed that companies were just as responsible for ethical and discretionary responsibilities as they were for economic outcomes. Moreover, although US consumers believed that companies had more responsibility for economic outcomes than philosophical or ethical endeavors, their scores on the latter (mean scores of 4.43 and 5.12 on a seven-point scale) indicate that they have moderately strong expectations for CSR. This previous research examined CSR more broadly than dealing with climate change and was focused on consumers rather than more general societal expectations, however, it does indicate that there are societal expectations for companies to be involved in CSR. Extrapolating from this, we propose that people in the community will also perceive that dealing with climate change is a corporation’s responsibility. In addition, rather than simply looking at mean scores, we also wanted to compare societal expectations for different loci of responsibility. To our knowledge, we are the first to compare loci of responsibility for dealing with climate change and therefore we do not have any evidence to suggest whether expectations for corporations are greater than that for other societal groups. Our first hypothesis, therefore, incorporates both our supposition that there will be high levels of expectations for corporations to deal with climate change and an exploratory examination of differences between societal groups:

Hypothesis 1: Community members will report strong expectations that it is a corporation’s duty to deal with climate change. We explore whether these expectations are greater than expectations that other societal groups (the international community, Federal, State and Local governments, individuals and families, and environmental groups) have a duty to deal with climate change.

### Factors Affecting Expectations of Corporations to Deal with Climate Change

Although large-scale studies often measure beliefs in issues such as climate change at a societal level, there will of course be variation amongst the people within that society. Moreover, factors that are related to this variation may act as trigger points for change. Therefore, beyond a description of the current social contract, our research also explores the factors that may affect people’s perception of the social contract regarding climate change.

Much of the research around individual-level climate change mitigation, particularly in the workplace, has examined environmental values and beliefs that climate change is real; this research generally shows significant relationships between a person’s pro-environmental mitigation actions and his or her beliefs or values (Bamberg and Moser, 2007; Unsworth et al., 2013; Young et al., 2013). In particular, belief in anthropogenic climate change is a key predictor [following the IPCC (2014)], we define belief in anthropogenic climate change as a belief that climate change is occurring and that it is caused by human activities). Although this occurs at the individual level of action, we suggest that a similar effect could occur when considering organizational actions. In other words, when a person believes in anthropogenic climate change they are also likely to believe that companies should engage in mitigation actions. Our reasoning is this. First, organizations are fundamentally comprised of concentrated human activity (Blackler, 1993). Second, if a person believes that climate change is caused by human activity then, assuming they are not anthropomorphizing organizations (e.g., Bruning and Galloway, 2003), climate change should be seen as being caused by the concentrated human activity existing within organizations. Third, and finally, if they see climate change as being caused by organizations, then they are more likely to hold organizations as responsible for dealing with it (Hamilton, 1978).

Moreover, research has shown that managers who see climate change as a threat to their organization are more likely to follow regulation rather than take on voluntary corporate environmental strategies (Sharma, 2000) therefore if a community member thinks that companies have a duty to deal with CSR, they may be unwilling to rely on managers engaging in it voluntarily (because if the manager sees it as a threat he or she will ignore the duty perceived by others) and instead are more likely to prefer that the company is held legally responsible. Thus, we propose that the more a person believes in anthropogenic climate change, the more likely they are to view climate change as a non-discretionary part of CSR (i.e., ethical or legal) and the more it is viewed as non-discretionary, the more likely it is to be seen as something that should be legally mandated; we therefore hypothesize that:

Hypothesis 2: Belief in anthropogenic climate change will be positively related to support for corporate regulatory policy (i.e., legal responsibility), mediated by a positive relationship with beliefs that corporations have a duty to deal with climate change (i.e., non-discretionary responsibility).

So far, we have followed the traditional thinking that belief in anthropogenic climate change (and its corollary for practical implication, changing people’s belief in anthropogenic climate change) will be a panacea for changing the public expectations related to a corporation’s responsibility for dealing with climate change. But will this be enough? We propose that as well as beliefs about climate change, when we consider the social contract with corporations we also need to consider beliefs about the perceived broader role of organizations in society; in other words, we suggest that free market ideology is necessary to consider.

An ideology is a worldview that is comprised of “a system of values, norms, and political preferences” (Carvalho, 2007, p. 225) and a free market ideology is defined as the “tendency to view market-based processes and outcomes not simply as efficient, but as inherently fair, legitimate, and just” (Jost et al., 2003, p. 55). It is ‘the invisible hand’ (Smith, 1776) that will take care of everything through market demand and competition.

Previous research has shown a main effect for free market ideology on belief in anthropogenic climate change; namely that it is significantly related to belief in anthropogenic climate change mediated by environmental apathy (Heath and Gifford, 2006), with stronger support for the free market corresponding to greater environmental apathy and in turn lower belief in anthropogenic climate change. However, we propose that free market ideology will have an additional effect to its relationship with anthropogenic climate change, and that is its effect on whether a person believes that dealing with climate change is a legal, ethical or discretionary responsibility of the organization. That is, we propose that free market ideology will affect a community member’s perception that a corporation should be held legally responsible for dealing with climate change. Logically, if an individual believes that the free market will solve the problem, that person should also hold the belief that the company is not directly responsible as the market will decide if the company needs to do anything; if consumers don’t want environmentally friendly products and services then why should the company have to provide them? Indeed, as noted earlier, Maignan (2001) found differences in consumer expectations of CSR across countries which she attributed to differing ideologies.

Empirical evidence for this can be seen in studies examining political orientation. Belief in the free market is a key differentiator of political orientations; those on the right (Conservatives, Republicans, Liberals) have strong beliefs in a key role for the free market and thus a weak role for government, while those on the left (Labor, Democrats, Greens) have less strong beliefs in the ability of the free market to overcome social and environmental problems (Heath and Gifford, 2006). Research has shown that those on the right tend to not only believe less in anthropogenic climate change (Dunlap and McCright, 2008; McCright, 2011; McCright and Dunlap, 2011) but, importantly, they are also less likely to support policies to mitigate climate change (Unsworth and Fielding, 2014).

**FIGURE 1:**
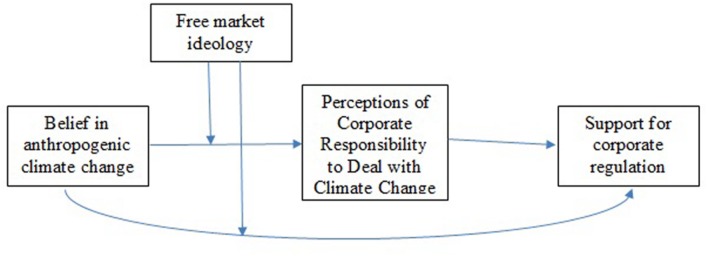
**Mediated moderation of climate change beliefs and free market ideology on corporate environmental responsibility and policy support**.

In addition, we argue that these two factors, belief in anthropogenic climate change and free market ideology, will interact. As discussed earlier, those who believe in anthropogenic climate change should, rationally, believe that all humans are responsible and thus that corporates (as human collectives) are also responsible for dealing with climate change; there should therefore be a positive relationship between anthropogenic climate change and belief in corporate responsibility to deal with climate change. However, if one has a strong free market ideology this relationship will be stronger than if one has a weak free market ideology because of the synergistic effects of free market ideology and anthropogenic climate change. In the first instance, the combination of a strong free market ideology and low belief in anthropogenic climate change will result in very low perceptions that a company has a duty to deal with climate change because the two beliefs reinforce each other; but having a strong free market ideology will not greatly affect the person’s perception of a corporation’s duty to deal with climate change if they have a high belief in anthropogenic climate change. This is because there is a strong logical relationship between believing that humans are responsible (high belief in anthropogenic climate change) and believing that companies, alongside others, are responsible for dealing with it. In the second instance, when free market ideology is weak, then a weak belief in anthropogenic climate change won’t be as detrimental to beliefs in corporate responsibility to deal with climate change and the resulting perception of corporate responsibility will not be as low; hence the relationship between anthropogenic climate change and a belief in corporate responsibility to deal with climate change will not be as strong. Overall, this means that the relationship between belief in anthropogenic climate change and perceptions that corporations have a duty to deal with climate change will be stronger when free market ideology is strong compared to when it is weak. Hypotheses 2 and 3 are illustrated in **Figure 1.**

Hypothesis 3: The mediated relationship between belief in anthropogenic climate change and support for regulatory policy via belief in corporate responsibility to deal with climate change will be moderated by free market ideology. This interaction is such that when free market ideology is strong, the mediated relationships will be stronger than when free market ideology is weak.

## Materials and Methods

### Sample

We used a large accredited research panel, Qualtrics, to access a wide range of participants from across Australia and across different within-nation subgroups. In total, 1066 respondents participated; of these, 50.1% were male and they ranged in age from 18 to 82 (mean age was 45.33 years, standard deviation was 15.12 years). Participants came from a range of educational backgrounds (8.5% left school at age 15, 22.0% had a high school education only, 36.7% had a trade qualification, 24.8% had a university Bachelor degree, and 8.1% had a Masters or Ph.D. qualification) and political orientations [35.3% supported for the Labor Party (mainstream center-left-wing), 32.6% supported the Liberal Party (mainstream right-wing), 5.0% supported The Nationals (rural right-wing), 10.6% supported The Greens (left-wing), and 16.6% supported Independents].

The study was carried out in accordance with the recommendations and approval of The University of Western Australia’s Ethics Committee. The survey was anonymous and no identifying information was collected. Informed consent was provided by participants clicking on a survey button and continuing to the survey after reading the online information sheet.

### Measures

We measured belief in anthropogenic climate change by asking participants, “How much do you think humans contribute to/cause climate change? (as a percent of overall climate change; if you do not believe that climate change is occurring, please answer ‘0’)?” Responses ranged from 0 to 100; the mean response was 54.77% and the standard deviation was 30.18%.

Free market ideology was measured using the five-item measure developed by Heath and Gifford (2006). Participants were introduced to the concept of a free-market before the questions. The items are: “The free-market system is likely to promote unsustainable consumption” (R); “The free-market system may be efficient for resource allocation, but it is limited in its capacity to promote social justice” (R); The preservation of the free market system is more important than localized environmental concerns; Free and unregulated markets pose important threats to sustainable development and “An economic system based on free-markets, unrestrained by government interference, automatically works best to meet human needs” and participants responded on a five-point scale from “Strongly Disagree” to “Strongly Agree.” The internal reliability of the scale was adequate (α = 0.67).

We measured the different loci of responsibility to deal with climate change by asking participants, “Whose responsibility is it to address climate change?” for seven different groups: National government, industry/companies, the international community, state governments, individual/families, local authorities, and environmental groups. Corporate environmental responsibility was captured with the item “Industry/companies.” Participants responded on a seven-point scale from “Not at all” to “Completely” for each locus of responsibility.

Finally, support for company regulatory policy was measured by asking participants the degree to which they supported a policy or policy option that could be used by a Federal Government which focused on “Stronger regulation of companies and their carbon emissions.” Participants responded on a five-point scale from “Strongly Disagree” to “Strongly Agree.”

## Results

Means, standard deviations, and correlations of the variables are presented in **Table 1.** As expected, there was a significant negative relationship between free market ideology and belief in anthropogenic climate change (*r* = -0.25, *p* < 0.001).

**Table 1:** Means, standard deviations, and correlations among key variables.

	Mean (*SD*)	1	2	3	4	5
1. Gender	1.50 (0.50)					
2. Age	45.33 (15.52)	-0.12ˆ***				
3. Belief in CC	54.77 (30.18)	0.11ˆ***	-0.07ˆ*			
4. Free market ideology	2.65 (0.64)	0.04	-0.14ˆ***	-0.25ˆ***		
5. Company env. responsibility	5.41 (1.78)	0.11ˆ***	0.05	0.51ˆ***	-0.32ˆ***	
6. Support for company regulation	3.86 (1.04)	0.08ˆ*	0.10ˆ**	0.40ˆ***	-0.34ˆ***	0.55ˆ***


*^∗^*p* < 0.05, ^∗∗^*p* < 0.01, ^∗∗∗^*p* < 0.001.*

In general support of our first hypothesis, and as can be seen in **Table 2**, on the whole, the community members in our sample believed that companies were to be held responsible for climate change (*M* = 5.41 on a seven-point scale). Only 26% of participants rated corporate environmental responsibility below the mid-way point on the scale. When comparing perceived corporate’s responsibility to deal with climate change to other loci of responsibility we found that people thought that companies were more responsible for dealing with climate change than the international community (*t* = 2.21, *df* = 1063, *p* < 0.05), the State Government (*t* = 5.64, *df* = 1063, *p* < 0.001), individuals and families (*t* = 17.61, *df* = 1063, *p* < 0.001), local authorities (*t* = 11.17, *df* = 1063, *p* < 0.001), and environmental groups (*t* = 12.97, *df* = 1063, *p* < 0.001) but there was no difference in perceived responsibility when compared with Federal Government (*t* = -0.34, *df* = 1063, *p* = 0.74). Thus, the Australian community believes that companies (together with National Governments) should address climate change.

**Table 2:** Means and standard deviations of the different locus of responsibilities.

Whose responsibility is it to address climate change?	Mean	Standard deviation
Industry/companies	5.41	1.78
National governments	5.42	1.78
International community	5.33	1.80
State governments	5.22	1.82
Individuals/families	4.64	1.94
Local authorities	4.96	1.86
Environmental groups	4.82	1.96


We assessed Hypothesis 2 using Model 4 in Hayes’ (2012) PROCESS Macro. Overall, there was support for our hypothesis. Belief in anthropogenic climate change was significantly related to the mediator, corporate responsibility to deal with climate change (*B* = 0.03, *SE(B)* = 0.002, *p* < 0.001), and the mediator was significantly related to company regulatory policy (*B* = 0.26, *SE(B)* = 0.02, *p* < 0.001) after controlling for gender and age. The hypothesized indirect effect of belief in anthropogenic climate change on regulatory policy via perceived corporate responsibility was significant at 0.008 (*SE* = 0.001, 95% CI [0.006, 0.009]). However, there was also a significant direct effect (0.006, *SE* = 0.001, *p* < 0.001, 95% CI [0.004, 0.008]) indicating partial mediation. In total, the model accounted for 32.6% of the variance in regulatory policy, *F*(4,1043) = 126.26, *p* < 0.001. We tested the robustness of the model by also including education and political orientation as control variables but these were not significantly related to support for regulatory policy when gender and age were included and did not affect the hypothesized relationships.

**FIGURE 2:**
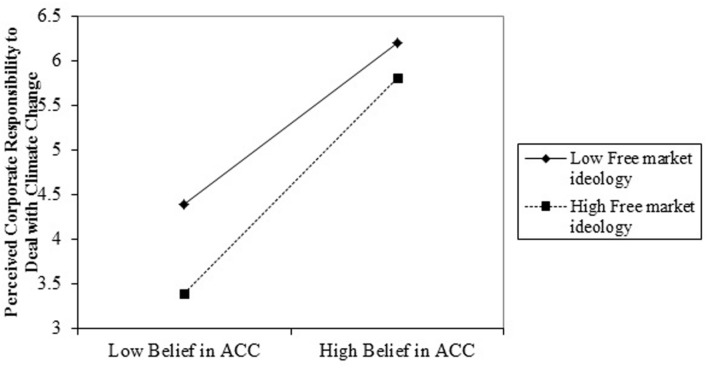
**Interaction between anthropogenic climate change beliefs (ACC) and free market ideology**.

To assess whether free market ideology affected this mediated relationship, we conducted a moderated mediated analysis using Model 8 of the PROCESS Macro (Hayes, 2012), using centered variables and controlling for age and gender. The results shown in **Table 3** indicate that although the perceived amount of human contribution was significantly related to perceived corporate responsibility to deal with climate change (*B* = 0.03, *SE(B)* = 0.002, *p* < 0.001, 95% CI [0.02, 0.03]), this was moderated by free market ideology (*B* = 0.01, *SE(B)* = 0.002, *p* < 0.001, 95% CI [0.003, 0.012]; **Figure 2**). Moreover, free market ideology had a strong direct effect on corporate responsibility to deal with climate change over and above the interaction (*B* = -0.53, *SE(B)* = 0.07, *p* < 0.001, 95% CI [-0.68, -0.38]). This moderation was also shown to affect support for ‘policy regulating companies’ carbon emissions indirectly via corporate responsibility to deal with climate change (*B* = 0.24, *SE(B)* = 0.02, *p* < 0.001, 95% CI [0.21, 0.27]), however, there was no direct effect of the moderation on support for company regulatory policy (*B* = 0.001, *SE(B)* = 0.001, *p* = 0.86, 95% CI [-0.002, 0.003]). Instead, there was a further strong negative relationship between free market ideology and support for regulatory policy (*B* = -0.27, *SE(B)* = 0.04, *p* < 0.001, 95% CI [-0.35, -0.18]). With regard to the conditional indirect effects, although indirect effects at high and low levels of free market ideology were significant and positive, the mediated indirect effect was strongest at high levels of free market ideology (0.008; 95% CI [0.006, 0.01]) and lowest at low levels of free market ideology (0.005, 95% CI [0.004, 0.007]), as expected.

**Table 3:** Results of the mediated moderation regression analysis.

	DV: company env. responsibility *B*(*SE*), *p*	DV: support for company regulation *B*(*SE*), *p*
Gender	0.26(0.09), *p* = 0.006	0.07(0.05), *p* = 0.17
Age	0.01(0.003), *p* = 0.002	0.004(0.001), *p* = 0.006
Belief in human contrib. to CC	0.03(0.001), *p* < 0.001	0.005(0.001), *p* < 0.001
Free market ideology	-0.53(0.07), *p* < 0.001	-0.27(0.04), *p* < 0.001
Belief in CC x free market	0.01(0.002), *p* < 0.001	0.0002(0.001), *p* = 0.87
Company env. responsibility		0.24(0.02), *p* < 0.001

Total	*R*^2^ = 0.32, *F*(5,1041) = 96.07, *p* < 0.001	*R*^2^ = 0.35, *F*(6,1040) = 93.43, *p* < 0.001


*B, unstandardized regression coefficient; SE, standard error of regression coefficient.*

Simple slopes analysis showed that although the positive relationship between anthropogenic climate change and perceived responsibility for companies to deal with climate change was significant at both high (one standard deviation above the mean) and low (one standard deviation below the mean) levels of free market ideology, it was much stronger when free market ideology was high (*t* = 4.09, *p* < 0.001; *t* = 2.22, *p* < 0.05; respectively). This is in line with our hypothesis that when free market ideology is strong, the mediated relationships should be stronger (because a weak belief in anthropogenic climate change will produce very low perceptions of corporate responsibility to deal with climate change and support for legal regulation) than when free market ideology is weak (because a weak belief in anthropogenic climate change won’t be as detrimental to belief in corporate responsibility to deal with climate change). An alternative way to interpret this interaction is to examine the simple slopes between free market ideology and perceived corporate responsibility to deal with climate change at different levels of anthropogenic climate change. This is statistically identical yet reveals additional understanding of the relationship. In this case, simple slopes analysis revealed that there was a significant negative relationship between free market ideology and corporate responsibility to deal with climate change at one standard deviation below the mean of anthropogenic climate change (*t* = -3.79, *p* < 0.001), but at the mean and at one standard deviation above the mean there was no significant relationship (*t* = -1.43, *p* = 0.15; *t* = -0.52, *p* = 0.60; respectively). To determine the point at which the relationship between free market ideology and perceived corporate responsibility was completely attenuated, we varied the percentage of human contribution to climate change and found that a non-significant relationship occurred at 45%; in other words, those who believed that less than 45% of climate change was due to human causes had a significant relationship between free market ideology and the perception that corporations were responsible for dealing with climate change.

Overall, the results suggest that both climate change views and free market ideology have a strong effect on perceptions that companies are responsible for dealing with climate change and on support for regulatory policy. Although focusing on increasing beliefs in anthropogenic climate change is important to help buffer the negative effects of free market ideology, particularly in perceptions of whether corporates are responsible for dealing with climate change, the strong direct effect between free market ideology and policy support and the absence of the intervening buffering effect of climate change beliefs means that free market ideology is a clear hindrance to implementing corporate regulatory policy.

## Discussion

In this research, we built on the ideas of Carroll (1991, 1999, 2000) and Donaldson and Dunfee (1994, 1999, 2000) and sought to understand the social contract underlying CSR, particularly with regards to climate change; namely, whether individuals in the community considered companies to be responsible for dealing with climate change and whether they would support government policy on regulating companies to do so. We argued that this was important to more fully understand our conceptualization of CSR. We found that people thought companies and government had a greater duty to deal with climate change compared to individuals/families, international community, and local associations; that the more an individual believed that humans contributed to climate change the more they held companies responsible to deal with climate change; but that those who believed in a free market were less likely to hold companies responsible or support regulatory policy particularly when they also did not believe in anthropogenic climate change.

We believe these findings are important from both a theoretical and a practical standpoint. Theoretically, most research that has examined antecedents of organizational-level CSR policies has focused on institutional and organizational factors and little empirical research has examined the role that individuals may play (Aguinis and Glavas, 2012; with the exception of individuals as consumers, see e.g., Russell et al., 2016). Of course, this was not explicitly multi-level research, in that we did not measure specific organizational reactions to individuals’ perceptions of responsibility, however, we believe that this adds to the growing field of research that is building the micro-foundations of CSR.

Moreover, we found that free market ideology is a substantial barrier to believing that companies have a responsibility to deal with climate change and supporting government policy toward that purpose. Previously, research on free market ideology has focused on the relationship between ideology and belief in anthropogenic climate change (e.g., Heath and Gifford, 2006) and ideology was assumed to be behind country differences in perceptions of CSR (Maignan, 2001). However, we argued that in addition to the mechanistic model (where free market ideology affects belief in climate change which then affects corporate responsibility beliefs), that free market ideology will play an important independent role as a moderator when we also consider the organizational context.

Indeed, we found this to be the case. We found that when free market ideology was weak then even a moderate level of belief in anthropogenic climate change would be associated with a perception that companies should deal with climate change. However, when free market ideology was strong then belief in anthropogenic climate change was very important. The combination of both strong free market ideology and little belief in anthropogenic climate change led to extremely low levels of perceived corporate responsibility to deal with climate change. Although we recognize that the effect size of this moderation is relatively low, given the importance of the topic and the multiplied error variance in moderation variables we believe that this is still an important finding.

A second finding, however, was that free market ideology was more central than we had originally thought. Although we predicted mediated moderation (where free market ideology moderates not only the relationship to the mediator, namely corporate responsibility, but also the relationship to the outcome variable, namely policy support), we found only moderated mediation. In other words, we found only a first stage moderation where free market ideology interacted with climate change beliefs on perceived corporate responsibility to deal with climate change (c.f., Langfred, 2004). We had also expected that free market ideology would interact with climate change beliefs to influence policy support, but instead we found only the indirect effect (via responsibility) and a direct main effect. Although this component of our hypothesis was not supported, we believe that it signals the strength of the effect of free market ideology. Even a strong belief in anthropogenic climate change is not able to moderate the effect of free market ideology on policy support. In other words, convincing people about anthropogenic climate change may result in increased perceptions of corporate responsibility even for those with a strong free market ideology and this may have some knock-on effect to policy support, but it will have only a limited impact on this outcome in buffering the overall effects of free market ideology.

Policy-makers therefore face an uphill battle in regulating organizations to be more environmentally responsible. Although the government was seen as just as responsible as corporations for dealing with climate change (presumably because of their ability to create policy), their task will not be easy. It is not enough to convince the community that climate change is real and that human activity is causing it. While this will help to some extent, its effect, particularly on policy, may well be limited. Those with a strong free market ideology will likely be those embedded within the capitalist system and potentially constitute a number of stakeholders both politically and organizationally. While some research has shown that demonstrating scientific consensus can counteract the negative effects of free market ideology on beliefs in climate change (Lewandowsky et al., 2013), it is unlikely that it will affect their views on policy support given the lack of interaction we found in our research. Instead, if regulatory environmental policy aimed at companies is desired, then other forms of engagement will be required.

It is important to acknowledge the limitations of our research and in doing so provide fruitful avenues for future research. The first limitation of our research is the cross-sectional nature of the design. This design was appropriate for our purpose of investigating the relationships in our study but we are unable to demonstrate causality between variables. It is not known, for example if a strong free-market ideology acts as an attention bias for scientific evidence on climate change. Such a bias may explain why there is a negative relationship between free-market ideology and belief in anthropogenic climate change. More knowledge of the direction of causality may enable future researchers and practitioners to design more effective campaigns to raise understanding and knowledge of anthropogenic climate change, and motivate future action.

The second limitation of research concerns the sample used in our study. Whilst this was a broad sample of Australian individuals and reflected the political diversity of the country, we must acknowledge the potential for culture to influence individuals’ perceptions regarding the implications of, and required action in response to, climate change (e.g., Lorenzoni and Pidgeon, 2006). Future research exploring the consistency of our findings in other countries, particularly those with more collectivist cultures or where environmental regulation of organizations is more stringent, would further our understanding of contextual contingencies and enable the design of more tailored campaigns. Finally, the measures used in our study displayed some limitations. The measure of anthropogenic climate change did not distinguish between those who did not believe in climate change at all and those who believed in naturally caused climate change; the measure of support for climate change regulation did not distinguish between those who supported any regulation and those who supported climate change regulation in particular; and the reliability of the free market ideology measure, while adequate, was not as high as one would ideally like.

Nonetheless, our research may be useful for policy makers and practitioners in their efforts to encourage future climate change action. Indeed, it may be that interventions designed to change behavior may need to ensure that they are concordant with the target’s ideology. Research suggests that goal concordance may be an important consideration in the success of pro-environmental behavior change interventions (Unsworth et al., 2013). In this way, for those individuals with a free market ideology it may be more important to appeal to economic goals and present a strong business case, rather than attempting to change their belief in anthropogenic climate change.

Our study reinforces the need to consider the social contract and, in particular, individual citizens and employees when examining the antecedents to organizational-level CSR (Carroll, 2004). The positive relationship between belief in anthropogenic climate change and beliefs that corporates are responsible for dealing with climate change and regulation to that end underlines the role that ordinary citizens may play in shaping the political and regulatory environment in which organizations operate. Our findings further illustrate the complexity of the challenge facing policy makers seeking to introduce environmental regulation, with free market ideology appearing to be a barrier to holding organizations responsible or supporting regulatory policy. This suggests that designing interventions and campaigns that pursue action on climate change will require multidisciplinary input (c.f., Kilbourne et al., 2002; Davis et al., 2014), e.g., from economists and political scientists as well as psychologists, in order to capitalize on non-environmental goals and present credible economic arguments that can appeal to those whose belief in the “invisible hand” is strong.

## Author Contributions

The study was designed and led by KU, with data collected as part of a wider project supported by a grant held jointly by KU and SR. KU led the data analysis and write-up. SR and MD contributed to data interpretation and manuscript drafting.

## Conflict of Interest Statement

The authors declare that the research was conducted in the absence of any commercial or financial relationships that could be construed as a potential conflict of interest.
